# Internet-Based Information Sharing With Families of Patients With Stroke in a Rehabilitation Hospital During the COVID-19 Pandemic: Case-Control Study

**DOI:** 10.2196/38489

**Published:** 2022-09-20

**Authors:** Tatsunori Murakami, Yumi Higuchi, Tetsuya Ueda, Wataru Kozuki, Aki Gen

**Affiliations:** 1 Graduate School of Rehabilitation Science Osaka Metropolitan University Habikino Japan; 2 Department of Physical Medicine and Rehabilitation Hoshigaoka Medical Center Japan Community Health Care Organization Hirakata City Japan

**Keywords:** COVID-19, internet-based, health information, stroke, family, rehabilitation, case-control, activities of daily living, information communication technology, physical function, recovery

## Abstract

**Background:**

The spread of COVID-19 has affected stroke rehabilitation. Given that inpatient visits are restricted in most institutions, alternative ways of providing information to family members are imperative. Informing families about patients’ rehabilitation progress via the web may help involve families in the rehabilitation process, enhance patients’ motivation to continue rehabilitation, and contribute overall to patients’ improvement in activities of daily living (ADL).

**Objective:**

We aimed to investigate the feasibility of the Internet-Based Rehabilitation Information Sharing (IRIS) intervention for families of patients with stroke at a rehabilitation hospital and examine the effect of IRIS on patients’ ADL improvement.

**Methods:**

In this case-control study, participants were inpatients at a rehabilitation hospital between March 2020 and April 2021. The intervention group (information and communication technology [ICT] group) included patients and families who requested IRIS, which consisted of a progress report on patients’ rehabilitation using text, photos, and videos. Those who did not receive internet-based information were included in the non-ICT group. The control group, matched with the ICT group based on a 1:1 propensity score, was selected from the non-ICT group. The covariates for calculating the propensity score were patients’ age, sex, and motor and cognitive scores on the Functional Independence Measure at admission. The main outcome was the degree of ADL improvement during hospitalization. Multiple regression analysis (forced entry method) was performed to confirm the impact of ICT use on ADL improvement. The independent variables were the presence of intervention, length of hospital stay, and number of days from onset to hospitalization.

**Results:**

In total, 16 groups of patients and families participated in the IRIS. The mean age of patients was 78.6 (SD 7.2) and 78.6 (SD 8.2) years in the ICT and control groups, respectively. The median total Functional Independence Measure difference was 28.5 (IQR 20.3-53.0) and 11.0 (IQR 2.8-30.0) in the ICT and control groups, respectively, and the ICT group showed significant improvement in ADL function (*P*=.02). In the multiple regression analysis of the ICT and control groups, the unstandardized regression coefficient was 11.97 (95% CI 0.09-23.84) for ICT use. These results indicate that ICT use was independently and significantly associated with improvement in ADL.

**Conclusions:**

This study examined the effect of IRIS on family members to improve ADL in patients with stroke who are hospitalized. The results showed that IRIS promotes the improvement of patients’ ADL regardless of age, sex, motor and cognitive functions at admission, and the length of hospital stay.

## Introduction

### Background

The spread of COVID-19 has affected medical institutions in general [1,2]. Given that inpatient visits are restricted in most institutions, communication between patients and families and between patients’ families and medical workers is difficult [3]. Studies have shown that family members of patients with stroke who are hospitalized are not usually informed about the type of care provided to patients, rehabilitation progress, and functional prognosis [4-6]. Therefore, if inpatient visits are restricted, alternative ways of providing information to family members are imperative [6].

Web-based interventions are expected to improve the quality of medical services [7]. There have been increasing reports on telemedicine and telerehabilitation. Web-based medical services include video conferencing, education for patients and their families, counseling, and rehabilitation support [8]. The expected benefits of the web in medical services are efficiency, convenience, and reduced COVID-19 infection risk due to noncontact [9]. In the future, web-based interventions will continue to be developed, and a variety of intervention methods and effects will be reported. However, at present, there are no previous studies using information and communication technology (ICT) to share information with family members of patients admitted to rehabilitation hospitals.

The guidelines for adult stroke rehabilitation published by the American Heart Association/American Stroke Association [10] indicate that “Communication and coordination between a large team, including the patient and his or her families, physicians, nurses, physical and occupational therapists, speech-language pathologists [is] paramount in maximizing the effectiveness and efficiency of rehabilitation. Without communication and coordination, isolated efforts to rehabilitate the stroke survivor are unlikely to achieve their full potential.” It is recommended that patient assessment in stroke rehabilitation be based on the International Classification of Functioning, Disability and Health [11]. Therefore, family information, such as family caregiving abilities and home environment, is useful for medical workers. Activities to intervene during hospitalization should be selected according to environmental factors after discharge. International Classification of Functioning, Disability and Health–based goals have been reported to increase rehabilitation efficiency [11]. With information from the family, personalized goals can be set for each patient.

Rehabilitation professionals have suspected that a patient’s motivation plays an important role in determining the outcome of therapy. Motivation for rehabilitation can be conceived as an internal “personality trait” of the individual patient, a quality that is affected by social factors [12]. It was reported that among patients in rehabilitation hospitals, those who perceive higher levels of family support are more motivated to improve mobility [13]. Therefore, the patient perception of family support is an important factor in enhancing the effectiveness of rehabilitation. However, during the COVID-19 outbreak, patients were isolated due to visitation limitations. Therefore, communication between patients and families mediated by health care providers is needed. Showing that family members care about the patient may improve their rehabilitation motivation.

### Objectives

Patients’ families play an important role in the rehabilitation of patients with stroke [14]. Health care providers should be more aware of the fact that a patient’s family acts as a facilitator and supporter of the patient’s functional improvement [15]. Therefore, interactive information sharing on rehabilitation with patients’ families via the web is expected to involve families’ ability to support rehabilitation, set personalized goals, motivate patients, and contribute to improving patients’ activities of daily living (ADL). Thus, we aimed to investigate the feasibility of the Internet-Based Rehabilitation Information Sharing (IRIS) intervention for families of patients with stroke at a rehabilitation hospital and examine the effect of IRIS on patients’ ADL improvement.

## Methods

### Study Design and Participants

In this case-control study, electronic medical records were retrospectively examined between March 2020 and April 2021. Participants were inpatients at a rehabilitation hospital in Hirakata City, Osaka, Japan. The inclusion criteria were being aged >60 years and having a stroke diagnosis. The exclusion criterion was having been institutionalized before stroke onset.

The study period was during a time when hospital policy restricted visiting for all patients following the spread of COVID-19 infection, making it difficult for family members to see the progress of rehabilitation. In this study, the intervention group (ICT group) included patients and families who requested internet-based information provision. Information forms placed at the reception counter of the wards were distributed to hospitalized patients, and posters were displayed in the corridors of the wards. The information sheet contained the email address of the physiotherapist in charge of the ward, and patients’ families could receive internet-based information by indicating their willingness to participate in the study by sending an email to the concerned physiotherapist.

In contrast, patients and families who did not receive internet-based information were included in the non-ICT group. The non-ICT group was selected as the control group after a 1:1 propensity score matching [16] with the ICT group. The covariates for calculating the propensity score were patients’ age, sex, motor score on the Functional Independence Measure (FIM) at admission, and cognitive score on the FIM. Nearest-neighbor matching was used as the matching method.

### Ethics Approval

This study was approved by the Research Ethics Committee at Osaka Prefecture University (2018-118) and the Research Ethics Committee at Japan Community Health Care Organization Hoshigaoka Medical Center (IRB-HG2146). Hoshigaoka Medical Center obtained consent from all patients for the use of anonymized data from patients who are hospitalized for clinical research. Patients were also offered the opportunity to opt out, and the information was posted on the hospital’s official website.

### Intervention

**Figure 1:**
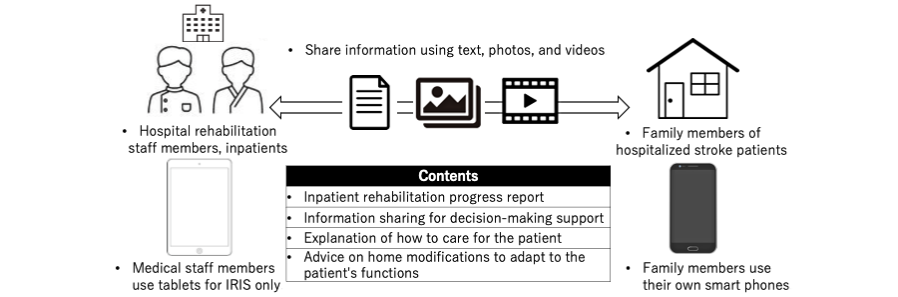
The overview diagram of Internet-Based Rehabilitation Information Sharing (IRIS).

The intervention, named IRIS, began within 2 weeks of admission to the rehabilitation hospital, and it was maintained until discharge. For the ICT group, the therapist reported on patients’ rehabilitation progress to their families at least once every 2 weeks using videos and text and responded to questions from family members. The IRIS mainly consisted of a progress report on rehabilitation. Videos were sent to patients practicing standing and walking during physiotherapy, patients practicing ADL during occupational therapy, and patients testing their higher brain functions in speech-language pathology. The intentions and concerns of patients’ families were also included to facilitate decision-making. Videos were used to explain ways to help assist a patient. Patients’ families sent pictures of their homes so that the physiotherapist in charge could suggest modifications to enable adaptation to patients’ functions. An overview of the IRIS is shown in Figure 1.

In addition, using IRIS, the medical staff interviewed the patient’s family members about the patient’s environmental factors and used these factors in setting the patient’s rehabilitation goals. For example, for a patient who lived alone during the day and had difficulty moving independently to the toilet, the patient’s family’s wishes were included when suggesting that the patient practice using a portable toilet. Additionally, in cases where the use of stairs was essential to enter the house, the need for stair climbing practice was identified and prioritized.

The information shared using IRIS between the health care provider and the patient’s family was also provided to the patient. The family learned about the patient’s rehabilitation process and informed the patient that the family expected the patient to improve. This information was used to encourage the patient’s rehabilitation motivation.

The Medical Care Station (MCS) application (Embrace Co., Ltd) was used to provide information. MCS is a security-conscious, “completely private” social networking service that shares information in a timeline format. Only authorized members can view MCS communications.

### Inpatient Rehabilitation

All patients received daily physical and occupational therapy, as well as speech and language therapy as needed. The total time commitment was a maximum of 3 hours per day. The program was determined by each in-charge therapist after evaluating the patient. The hospital held a face-to-face conference approximately 1 month after admission, and the medical staff informed patients’ families about the rehabilitation progress during their hospital stay.

### Main Outcome

The main outcome was the degree of ADL improvement during hospitalization. The total FIM scores at the time of admission and discharge were evaluated by the occupational therapist in charge, and the difference between the 2 scores was defined as ADL improvement. In addition, data on patients’ age, sex, discharge destination, hospital stay, and the number of days from onset to hospitalization were obtained from the medical records.

### Characteristics of the Patient’s Family

In the ICT group, patient family information, including age, sex, relationship with the patient (spouse or child), and occupation, were obtained from the MCS.

### Statistical Analysis

For univariate comparisons, the *χ*^2^ test, uncorrelated 2-tailed *t* test, and Mann-Whitney *U* test were used. Multiple regression analysis (forced entry method) was performed to confirm the impact of ICT use on ADL improvement. In the analysis of the ICT and control groups, the independent variables were the presence of intervention, length of hospital stay, and number of days from onset to hospitalization.

## Results

### Control Group Selection

**Figure 2:**
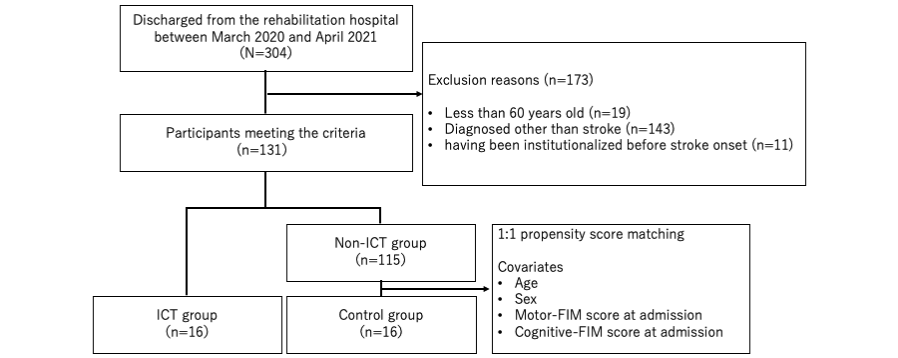
Flow of the study participants. FIM: Functional Independence Measure; ICT: information and communication technology.

A total of 131 participants met the inclusion criteria during the study period. The patient results are presented in Table 1. In total, 16 groups of patients and families participated in the IRIS. The median (IQR) total FIM score on admission was 37.0 (28.8-79.3) in the ICT group and 75.0 (55.0-97.0) in the non-ICT group, and patients who received IRIS reported significantly lower independence in ADL on admission (*P*=.004). In addition, 16 control participants were selected from the non-ICT group and matched to the ICT group using age, sex, motor FIM score at admission, and cognitive FIM score at admission as covariates (Figure 2). The total FIM score at admission for the control group was 40.5 (IQR 22.8-81.8), which was not significantly different from that of the ICT group (*P*=.90).

**Table 1:** Comparison of ICT and control groups by matching. *P* values are all based on comparisons with the ICT group.

Variable		ICT^a^ group (n=16)	Control group (n=16)	*P* value	Non-ICT group (n=115)	*P* value
Age (years), mean (SD)		78.6 (7.2)	78.6 (8.2)	.96^b^	77.3 (8.2)	.53^b^
Sex, female, n (%)		6 (37.5)	5 (31.2)	>.99^c^	50 (43.5)	.79^c^
**Type of stroke, n (%)**				.45^c^		.81^c^
	Ischemic	10 (62.5)	7 (43.8)		79 (68.7)	
	Hemorrhagic	5 (31.2)	6 (37.5)		32 (27.8)	
	Subarachnoid hemorrhage	1 (6.2)	3 (18.8)		4 (3.5)	
Discharge destination, home, n (%)		10 (62.5)	8 (50)	.72^c^	85 (73.9)	.38^c^
Length of hospital stay (days), mean (SD)		95.3 (37.4)	70.6 (33.5)	.06^b^	66.0 (37.3)	.004^b^
Onset to hospitalization (days), mean (SD)		31.4 (17.5)	30.9 (15.3)	.93^b^	27.7 (15.7)	.38^b^
**Total FIM^d^ score (out of 126), median (IQR)**						
	Admission	37.0 (28.8-79.3)	40.5 (22.8-81.8)	.90^e^	75.0 (55.0-97.0)	.004^e^
	Discharge	81.0 (60.3-105.0)	62.5 (29.5-109.5)	.21^e^	105.0 (78.0-119.0)	.03^e^
	Difference	28.5 (20.3-53.0)	11.0 (2.8-30.0)	.02^e^	22.0 (10.0-31.0)	.02^e^
**Motor FIM score (out of 91), median (IQR)**						
	Admission	21.5 (13.0-51.3)	20.5 (13.0-53.0)	.90^e^	51.0 (31.0-66.0)	.003^e^
	Discharge	64.0 (38.8-76.3)	42.5 (16.0-80.0)	.14^e^	79.0 (54.0-87.0)	.06^e^
	Difference	24.0 (17.0-46.0)	10.5 (3.0-25.8)	.03^e^	20.0 (9.0-27.0)	.048^e^
**Cognitive FIM score (out of 31), median (IQR)**						
	Admission	19.0 (12.3-26.5)	20.0 (9.3-28.5)	.93^e^	26.0 (20.0-32.0)	.01^e^
	Discharge	22.0 (16.3-29.0)	21.0 (11.3-30.0)	.78^e^	28.0 (21.0-33.0)	.02^e^
	Difference	2.0 (0.0-5.0)	0.0 (0.0-1.8)	.29^e^	0.0 (0.0-3.0)	.28^e^

^a^ICT: information and communication technology.

^b^Unpaired 2-tailed *t* test.

^c^*χ*^2^ test.

^d^FIM: Functional Independence Measure.

^e^Mann-Whitney *U* test.

### Main Outcome

The mean age of the patients was 78.6 (SD 7.2) and 78.6 (SD 8.2) years in the ICT and control groups, respectively. The median total FIM difference, which was the main outcome, was 28.5 (IQR 20.3-53.0) and 11.0 (IQR 2.8-30.0) in the ICT and control groups, respectively, and the ICT group showed significant improvement in ADL function (*P*=.02). The mean number of days from onset to hospitalization was 31.4 (SD 17.5) and 30.9 (SD 15.3) days for the ICT and control groups, respectively, which was not significantly different (*P*=.93). The length of hospital stay—95.3 (SD 37.4) and 70.6 (SD 33.5) days for the ICT and control groups, respectively—showed a trend toward a longer hospital stay, but this result was not statistically significant (*P*=.06).

In the multiple regression analysis of the ICT and control groups, the unstandardized regression coefficients were 11.97 (95% CI 0.09-23.84) for ICT use, 0.19 (95% CI 0.03-0.36) for the length of hospital stay, and –0.37 (95% CI –0.72 to –0.02) for the number of days from onset to hospitalization. These results indicate that ICT use was independently and significantly associated with ADL improvement (see Table 2).

**Table 2:** Factors associated with activities of daily living improvement. Multiple regression analysis (forced imputation method) was used, with the dependent variable being the total Functional Independence Measure difference (R2=0.420). The number of participants is the sum of those in the ICT^a^ and control groups (n=32).

Factor	B	*β*	95% CI	*P* value
Group (1: ICT group)	11.97	0.32	0.094-23.840	.048
Length of hospital stay (days)	0.19	0.37	0.031-0.356	.02
Onset to hospitalization (days)	–0.37	–0.31	–0.719 to –0.018	.04

^a^ICT: information and communication technology.

### Characteristics of the Patient’s Family

Information on the 16 family members in the ICT group is as follows. The 16 family members of the ICT group included 2 (12%) spouses (aged 64 and 74 years; both were female) and 14 (88%) children (6 sons and 8 daughters). There were 2 (12%) children in their 30s, 7 (44%) in their 40s, and 5 (31%) in their 50s (Table 3). Additionally, 13 (81%) family members were working.

No adverse events, such as information leakage, were reported.

**Table 3:** Families’ characteristics in the information and communication technology group.

Characteristic		Family member (n=16), n (%)
**Age (years)**		
	30-39	2 (12)
	40-49	7 (44)
	50-59	5 (31)
	60-69	1 (6)
	70-79	1 (6)
**Employment status**		
	Working	13 (81)
	Not working	3 (19)

## Discussion

### Principal Findings

This study examined the effect of IRIS on family members to improve the ADL of patients with stroke who are hospitalized. The results showed that IRIS promoted patients’ ADL improvement regardless of age, sex, motor and cognitive function at admission, or the length of hospital stay.

### Comparison to Prior Work

In a previous study, a 4-day consecutive empowerment program for family members of patients with stroke who are hospitalized showed an improvement trend in patients at 2 weeks (during hospitalization) and significant improvement in patients’ ADL at 2 months (after discharge) compared to that of the control group [17]. Therefore, interventions for family members of patients with stroke who are hospitalized may indirectly improve the patients’ ADL. During the study period, inpatient visits were restricted to prevent the spread of COVID-19, so the control group families may have been less well informed than usual [18]. Therefore, we believe that the IRIS is an effective way to provide information, encourage the involvement of patients’ families in the rehabilitation process, and improve the effectiveness of the rehabilitation program.

In the Cochrane Database, there is some very low–quality evidence that goal setting may improve some outcomes for adults receiving rehabilitation for an acquired disability [19]. Personalized care planning leads to improvements in certain indicators of physical and psychological health status [20]. By obtaining more information on environmental factors from the patient’s family, IRIS was able to set personalized and appropriate goals, which may have contributed to improved patient functioning.

Living alone has been reported to be the strongest predictor of poststroke depression [21]. Living alone has also been reported to be a factor that leads to worse outcomes in inpatient rehabilitation after stroke. Furthermore, it was reported that the outcome of inpatient rehabilitation was better for those who lived with their children than for those who lived only with their spouses. This result was not due to differences in the time from stroke onset to hospital arrival [22], which may indicate that functional recovery was more effective in rehabilitation for patients with cohabiting family members than those living alone, who were less likely to have recovery expectations from their family members. There is evidence that information improves patient and caregiver knowledge about stroke and reduces patient depression scores [23]. Additionally, feeling strongly supported by family members has been reported to improve motivation to exercise [24]. It is possible that communicating the patient’s family’s recovery expectations to the patient through the IRIS program may have improved the patient’s motivation for rehabilitation and promoted ADL improvement.

Strokes often occur around the age of 70-80 years [25], and children of patients with stroke are often aged 40-50 years. Many family members support patients while working, but their busy schedules may make it difficult to both work and support the patients [26]. Moreover, documents related to inpatient care are difficult to understand and may stress patients’ families. The web, however, makes it possible to share videos and thereby transmit information that is visually easy to understand. Patients’ families do not need to worry about the time of the day when checking or sending information. Furthermore, the system can be used as a measure to restrict inpatient visits to prevent the spread of COVID-19. In the future, information sharing between hospital staff and patients’ families using the web will likely become more common.

### Strengths and Limitations

This study is the first to examine IRIS for family members of patients with stroke who are hospitalized to improve patients’ ADL. Despite the importance of these findings, this study had a few limitations. First, this study was not a randomized controlled trial. Since we were not involved in the allocation process, we were unable to eliminate the effects of selection bias and unmeasured confounders. Family members of patients who want to share information via the web may be more likely to be proactive in supporting patients. Previous studies have reported that factors associated with ADL improvement in patients with stroke who are hospitalized include patients’ age [27], sex [28], and motor and cognitive functions at the time of admission [29]. In this study, patients’ age, sex, and motor function and cognitive function at admission were used as covariates for propensity score matching and selecting a control group. We also used the length of stay, which is an indicator of the degree of the rehabilitation provided, as a covariate in the multiple regression analysis. Therefore, although this study was not a randomized controlled trial, we believe that we statistically adjusted for the confounders as much as possible. Second, limited information on patients’ families was collected. In the future, we plan to investigate family members’ understanding of patients, satisfaction with inpatient care, and psychological anxiety. Third, we were unable to evaluate patients’ motivation for rehabilitation. It is unclear whether sharing information with patients’ families via the web affects patients’ motivation. Fourth, the study was conducted at a single institution, and the sample size was small. Therefore, it is necessary to conduct a large-scale study to verify the generalizability of the study findings.

### Future Research Directions

Considering the difficulty of providing information for patients’ families due to inpatient visitation restrictions owing to COVID-19, we believe that the IRIS has a reasonable demand. Additionally, patients’ families’ responses to the IRIS were positive, and no adverse events were reported. Thus, it would be useful to continue implementing the IRIS to disseminate vital patient-related information to patients’ families.

## Abbreviations

ADL: activities of daily living
FIM: Functional Independence Measure
ICT: information and communication technology
IRIS: Internet-Based Rehabilitation Information Sharing
MCS: Medical Care Station

## Data Availability

The data sets analyzed during the current study are available from the corresponding author on reasonable request.
